# SCUDDO: an unsupervised clustering algorithm for single-cell Hi-C maps using diagonal diffusion operators

**DOI:** 10.1093/bioinformatics/btag284

**Published:** 2026-05-11

**Authors:** Luka Maisuradze, Mark D Shattuck, Corey S O’Hern

**Affiliations:** Department of Molecular Biophysics and Biochemistry, Yale University, New Haven, CT 06520, United States; Benjamin Levich Institute and Physics Department, The City College of New York, New York, NY 10031, United States; Department of Mechanical Engineering, Yale University, New Haven, CT 06520, United States; Graduate Program in Computational Biology and Biomedical Informatics, Yale University, New Haven, CT 06520, United States

## Abstract

**Motivation:**

Advances in high-throughput chromatin conformation capture have provided insight into the three-dimensional structure and organization of chromatin. While bulk Hi-C experiments capture spatio-temporally averaged chromatin interactions across millions of cells, single-cell Hi-C experiments report on the chromatin interactions of individual cells. Supervised and unsupervised algorithms have been developed to embed single-cell Hi-C maps and identify different cell types. However, single-cell Hi-C maps are often difficult to cluster due to their high sparsity, with state-of-the-art algorithms achieving a maximum Adjusted Rand Index (ARI) of only ≲0.4 on several datasets.

**Results:**

We introduce a novel unsupervised algorithm, Single-cell Clustering Using Diagonal Diffusion Operators (SCUDDO), to embed and cluster single-cell Hi-C maps. We evaluate SCUDDO on four previously difficult-to-cluster single-cell Hi-C datasets, and show that it can outperform other current algorithms in ARI by ≳0.2. Further, SCUDDO outperforms all other tested algorithms even when we restrict the number of intrachromosomal maps for each cell type and when we use only a small fraction of contacts in each Hi-C map. Thus, SCUDDO can capture the underlying latent features of single-cell Hi-C maps and provide accurate labelling of cell types even when cell types are not known *a priori*.

**Availability and implementation:**

SCUDDO is freely available at https://www.github.com/lmaisuradze/scuddo as well as https://doi.org/10.6084/m9.figshare.31759915. The tested datasets are publicly available and can be downloaded from the Gene Expression Omnibus.

## 1 Introduction

Elucidating the structure and dynamics of chromatin in cell nuclei is essential for understanding numerous cellular processes such as DNA transcription and replication (Misteli 2020). Advances in whole-genome analyses, e.g. chromosome conformation capture techniques such as Hi-C, have provided important insights into long-range chromatin interactions and hierarchical chromatin organization (Lieberman-Aiden et al. 2009). Hi-C experiments provide chromatin contact maps, often represented as a symmetric matrix A, where $A_{ij}$ is the number of times that loci *i* and *j* of chromatin come into close proximity. Bulk Hi-C contact maps provide information on chromatin fragment interactions averaged over millions of cells. In contrast, *single-cell* Hi-C maps give the frequency of chromatin contacts in each individual cell.

It is now well established that chromatin structure and organization can differ significantly across cell populations (Finn et al. 2019, Misteli 2020). Transcription analyses and imaging studies have shown that gene expression profiles and cell morphology can differ even between genetically identical cells (Shah et al. 2018, Finn et al. 2019, Rodriguez et al. 2019). In addition, the size and location of chromatin loops and topologically associating domains (TADs) can vary between the Hi-C maps of individual cells for a given organism (Cattoni et al. 2017, Bintu et al. 2018, Finn and Misteli 2018, Mateo et al. 2019). As a result, the loci that possess high contact frequencies in bulk Hi-C maps can differ from those that are in close spatial proximity in fluorescence *in situ* hybridization (FISH) experiments, in part due to the heterogeneity in chromatin structure across individual cells (Bickmore and van Steensel 2013, Williamson et al. 2014, Giorgetti and Heard 2016, Fudenberg and Imakaev 2017, Shi and Thirumalai 2019). Thus, bulk Hi-C maps cannot be used to capture the structure and organization of chromatin in individual cells.

**Figure 1:**
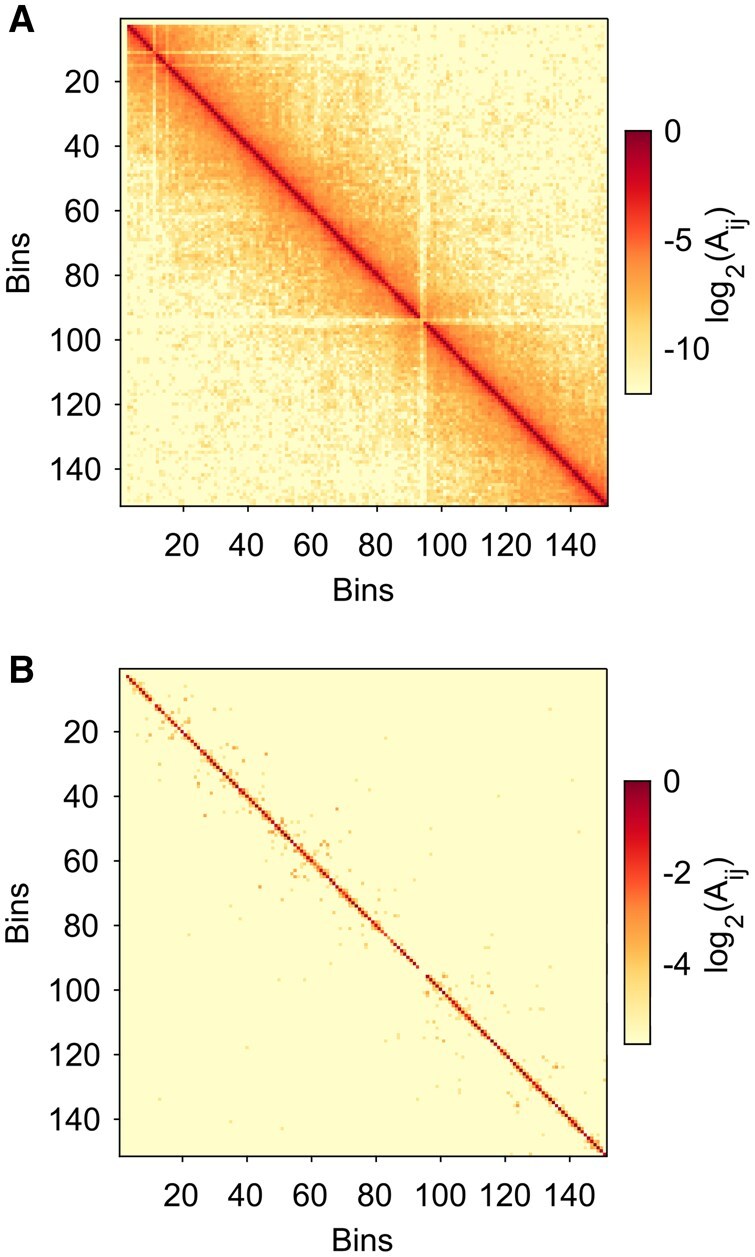
(A) A pseudo-bulk Hi-C map (${\;\text{log}\;}_{2}A_{ij}$ where zero counts are rendered with the colormap's minimum color) for chromosome 5 using pooled mouse oocyte cells before division from the Collombet dataset (Collombet et al. 2020) (normalized so that $\text{max}(A_{ij})=1$). (B) An example single-cell Hi-C map from the Collombet dataset for chromosome 5 of a mouse oocyte cell using the same normalization.

Several single-cell Hi-C technologies have been developed to capture chromatin interactions for large numbers of individual cells (Nagano et al. 2013, 2017, Flyamer et al. 2017, Stevens et al. 2017, Li et al. 2019). Single-cell Hi-C techniques enable studies of genome organization in individual cells, as well as comparisons of chromatin structure and organization across different cell types. Using data from single-cell Hi-C experiments, computational studies have focused on TAD, loop, and compartment identification for individual cells within a population (Flyamer et al. 2017, Stevens et al. 2017). However, despite the rapid advances in genome-wide assays, single-cell Hi-C maps are still sparse, only capturing a fraction of the interactions that are obtained in bulk Hi-C experiments (Galitsyna and Gelfand 2021, Zhen et al. 2022). For example, in Fig. 1, we show a pseudo-bulk Hi-C map for chromosome 5 in mouse oocyte cells (Collombet et al. 2020) and compare it to a single-cell Hi-C map for the same chromosome and cell type. The single-cell Hi-C map shows significant sparsity, with most of the off-diagonal elements having a count of 0, as well as large variability for elements near the diagonal.

While techniques like fluorescence-activated cell sorting can be used to label single cells during chromosome conformation capture methods, these techniques are more expensive, lower throughput, and not as widely available as single-cell Hi-C experiments. Thus, the development of classification algorithms for single-cell Hi-C maps may enable researchers to identify the key chromatin interactions that distinguish different cell types. Algorithms developed for bulk Hi-C analysis, including topologically associating domain callers (Maisuradze et al. 2024), often work with limited efficacy on raw single-cell Hi-C data. Thus, due to their inherent variability and sparsity, specialized algorithms must be developed to identify robust features in single-cell Hi-C maps. In this article, we focus on the specific task of classifying single-cell Hi-C maps based on the cell labels that have been provided by experimental studies. Most algorithms for clustering single-cell Hi-C maps use dimensionality reduction, treating each single-cell Hi-C map as a point in high-dimensional space and then mapping each point to a lower-dimensional space to cluster the data (Liu et al. 2018, Galitsyna and Gelfand 2021, Zhen et al. 2022). Despite the fact that there are more than a dozen algorithms to date for clustering single-cell Hi-C maps, there are many single-cell Hi-C datasets for which these methods achieve a maximum adjusted Rand index ARI≲0.4 (Zhen et al. 2022, Zheng et al. 2022). Moreover, there are many cases where one clustering method performs well on one single-cell Hi-C dataset, but then performs poorly on another dataset (Galitsyna and Gelfand 2021, Zhen et al. 2022), suggesting that current methods have trouble identifying features that generalize across multiple single-cell Hi-C datasets for clustering.

We develop a novel algorithm, single-cell clustering using diagonal diffusion operators (SCUDDO), which is fully unsupervised, fast, and easy to interpret to separate single-cell Hi-C maps into distinct groups. We then compare the predicted labels of the single-cell Hi-C maps to the cell types that are provided by experimental studies. To quantify the accuracy of the clustering, we calculate the ARI and normalized mutual information (NMI) using the predicted and ground truth labels. We find that SCUDDO outperforms current state-of-the-art methods on four difficult-to-cluster single-cell Hi-C datasets, achieving an ARI and NMI greater than those for all of the tested methods on each of the datasets. We also find that SCUDDO achieves higher accuracy for clustering single-cell Hi-C maps compared to other algorithms when using only a fraction of the number of intrachromosomal maps and a fraction of the superdiagonals in each map.

The remainder of the manuscript is organized as follows. In the Materials and Methods section, we describe the key elements and hyperparameters of SCUDDO for clustering single-cell Hi-C maps. We then describe the four difficult-to-cluster datasets for benchmarking SCUDDO and the two metrics (ARI and NMI) for quantifying the clustering accuracy. In the Results section, we provide the ARI and NMI scores for SCUDDO and five current methods for clustering single-cell Hi-C maps on each of the four difficult-to-cluster Hi-C datasets. We also show SCUDDO’s performance across different hyperparameter regimes and when limiting the number of superdiagonals and intrachromosomal maps sampled. In the Discussion, we include some interpretations of the results, our conclusions, and promising future research directions.

## 2 Materials and methods

**Figure 2:**
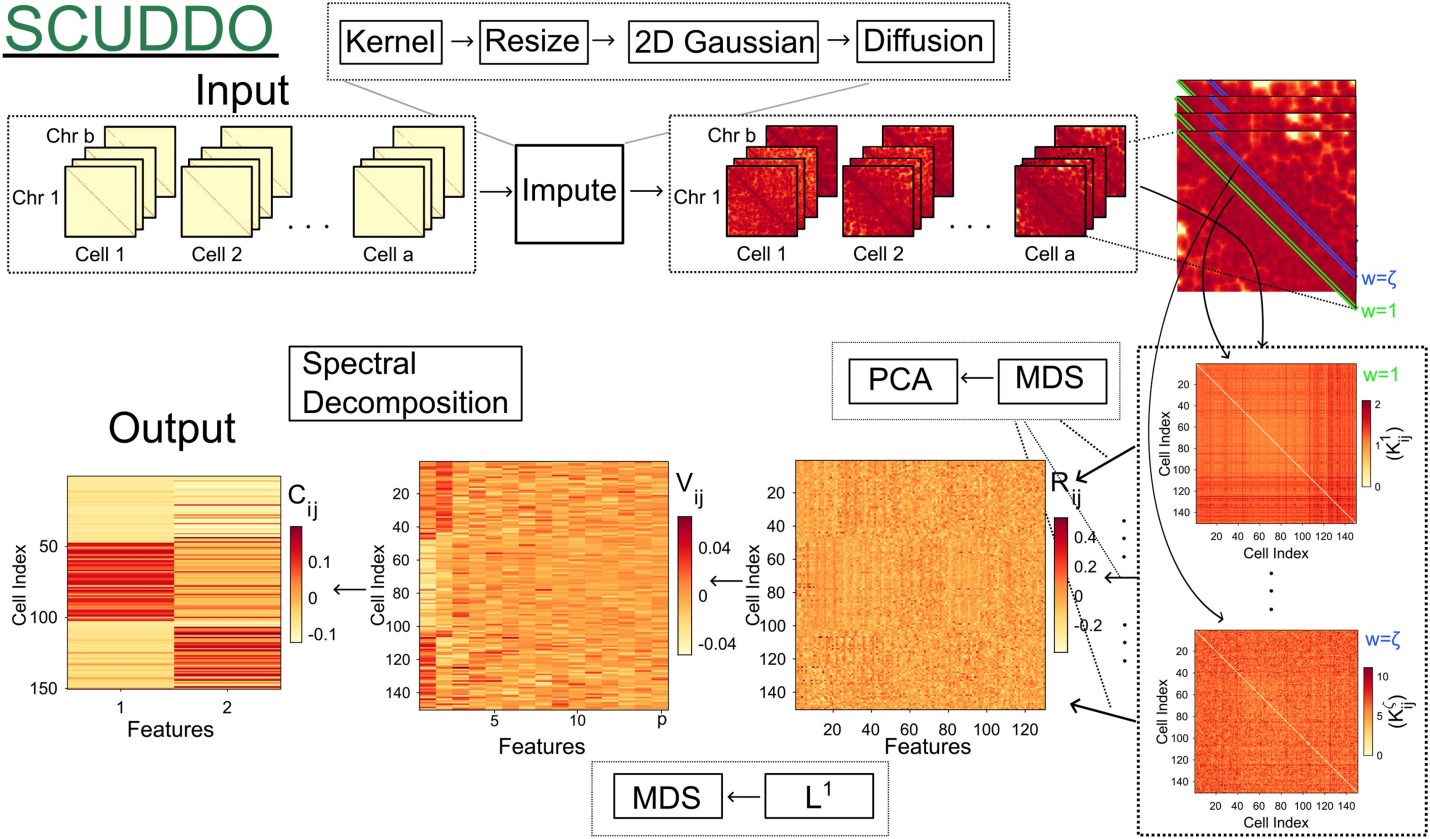
A schematic of the SCUDDO algorithm for clustering single-cell Hi-C maps. SCUDDO first imputes the set of intrachromosomal Hi-C matrices (indexed by $A_{s}^{k}$) for each cell and then samples each superdiagonal (indexed by *w*) from each Hi-C matrix to form a feature matrix $K^{w}$ for each sampled superdiagonal. Principal component analysis (PCA) and nonmetric MDS are then applied to each feature matrix to form the matrix R, which is then embedded in a lower-dimensional latent space using the $L^{1}$ norm to form the embedding, V, which is then finally spectrally decomposed into C.

The Materials and Methods is organized into three subsections. We first define the necessary notation and summarize the steps of SCUDDO to cluster single-cell Hi-C maps (see Figure 2 for a summary of SCUDDO algorithm). Second, we describe four difficult-to-cluster single-cell Hi-C datasets that will be used to benchmark SCUDDO alongside five other single-cell Hi-C clustering algorithms. Finally, we define the two metrics, ARI and NMI, which are used to quantify clustering accuracy.

### 2.1 SCUDDO algorithm

The SCUDDO algorithm takes as input a set of intrachromosomal Hi-C maps with nonnegative integer entries for *a* cells, each with *b* chromosomes, totaling a×b intrachromosomal Hi-C maps. As for bulk Hi-C maps, single-cell Hi-C maps are represented as symmetric matrices with elements $A_{ij}$ that represent the number of contacts between loci *i* and *j* on chromatin. To distinguish between the cell and chromosome indices, we define $A_{s,ij}^{k}$ as the ijth element of the $n^{k}\times n^{k}$ Hi-C map for chromosome *k* of cell *s*. $n^{k}$ only depends on *k* since the dimensions of the Hi-C map only vary across different chromosomes. Given a set of intrachromosomal matrices, SCUDDO returns a low-dimensional embedding of the Hi-C matrices. This embedding is then used as an input into a clustering algorithm, e.g. K-means++ (Arthur and Vassilvitskii 2007), where each cell is assigned to one of *l* predicted labels.

SCUDDO starts by pre-processing and performing imputation on each intrachromosomal matrix $A_{s}^{k}$. First, SCUDDO checks each chromosome’s intrachromosomal matrices for any zero (empty) matrices and discards all intrachromosomal matrices for a chromosome if greater than ten percent are empty. Next, SCUDDO calculates a version of the regularized graph Laplacian kernel (Smola and Kondor 2003) for each $A_{s}^{k}$:


(1) $${\bar{A}}_{s}^{k}={\left(2I-D^{-1}(A_{s}^{k}+I)\right)}^{-1},$$

where *I* is the identity matrix, and *D* is the degree matrix of $A_{s}^{k}+I$. Then, each ${\bar{A}}_{s}^{k}$ is symmetrized and then reshaped into a r×r matrix, $A_{s}^{\prime k}$, using a bicubic interpolation kernel, where $r=\sum_{k=1}^{b}n^{k}/b$. SCUDDO then convolves each intrachromosomal matrix with a Gaussian kernel:


(2) $$A_{s,ij}^{\prime \prime k}=\sum_{\chi =1}^{3}\sum_{\omega =1}^{3}G_{\chi \omega }A_{s,(i-1+\chi )(j-1+\omega )}^{\prime k},$$

where G is a two-dimensional 3×3 Gaussian kernel with standard deviation σ=0.5 that uses replicate padding, where values outside of the bounds of the original Hi-C map are set to the values of the nearest border entry (for more details see Fig. 1, available as supplementary data at *Bioinformatics* online). G smooths local regions in each individual intrachromosomal matrix. The final pre-processing step is to normalize each intrachromosomal matrix and apply a matrix exponential:


(3) $$B_{s}^{k}=e^{\left(-\frac{A^{\prime \prime }{\;}_{s}^{k}}{\sum_{ij}A^{\prime \prime }{\;}_{s,ij}^{k}}\right)},$$

where $e^{A}$ denotes the matrix exponential, the convergent power series defined for any square matrix *A* as: $e^{A}=\sum_{k=0}^{\infty }\frac{1}{k!}A^{k}$. In the context of continuous-time network dynamics, $e^{tA}$ serves as a time-evolution operator, where *t* dictates the duration and scale of information propagation across the network. While positive values of *t* model forward diffusion, which smooths the data and spreads the signal across the network, setting t=−1 as SCUDDO does yields a dissipative system. We suspect that this inverse operation functions as a spectral high-pass filter where rather than blurring structural features, it penalizes indirect transitive paths and sharpens the local topological boundaries of the contact map. Next, we construct high-dimensional embeddings of the intrachromosomal Hi-C maps for each cell. Let $d^{w}(B_{s}^{k})$ be the ordered set of Hi-C map entries on the *w*th superdiagonal of the r×r matrix $B_{s}^{k}$:


(4) $$d^{w}(B_{s}^{k})=\{B_{s,1,1+w}^{k},B_{s,2,2+w}^{k},\ldots ,B_{s,r-w,r}^{k}\}.$$

For a given *w*, $d^{w}(B_{s}^{k})$ for each chromosome *k* for cell *s* is concatenated to form the embedding vector:


(5) $${\vec{e}}_{s}^{\;w}=\{d^{w}(B_{s}^{1}),d^{w}(B_{s}^{2}),\ldots ,d^{w}(B_{s}^{b})\},$$

where α=1,…,b(r−w) indexes the entries in ${\vec{e}}_{s}^{\;w}$. Every embedding vector, ${\vec{e}}_{s}^{\;w}$, is z-score normalized such that


(6) $${\vec{e}}_{s}{\;}^{\prime w}=\frac{{\vec{e}}_{s}^{w}-\mu }{\sqrt{\frac{1}{b(r-w)-1}\sum_{\alpha =1}^{b(r-w)}{\left({\left({\vec{e}}_{s}^{w}\right)}_{\alpha }-\mu \right)}^{2}}},$$

where $\mu =\sum_{\alpha =1}^{b(r-w)}{\left({\vec{e}}_{s}^{\;w}\right)}_{\alpha }/[b(r-w)]$. This pooling approach is similar to previous work (Zheng et al. 2022, Fletez-Brant et al. 2024) that employs band normalization for Hi-C matrices. Next, each embedding vector is transformed into a signed difference vector:


(7) $${\vec{f}}_{s}^{w}=\text{sgn}(\nabla (\vec{e}{\;}_{s}^{\prime \;w})),$$

where $\nabla {\left(\vec{e}{\;}_{s}^{\prime \;w}\right)}_{\alpha }={\left(\vec{e}{\;}_{s}^{\prime \;w}\right)}_{\alpha }-{\left(\vec{e}{\;}_{s}^{\prime \;w}\right)}_{\alpha +1}$ and sgn is the sign function. (Note that we set the last entry of the chromosome difference vector ${\left({\vec{f}}_{s}^{\;w}\right)}_{b(r-w)}={\left(\vec{e}{\;}_{s}^{\prime \;w}\right)}_{b(r-w)}$.) Eq. 7 transforms $\vec{e}{\;}_{s}^{\prime \;w}$ into a ternary vector with values 1, 0, or −1. For a given *w*, each cell’s difference vector, ${\vec{f}}_{1}^{\;w},{\vec{f}}_{2}^{\;w},\ldots ,{\vec{f}}_{a}^{\;w}$ is used to calculate the distance matrix between cells *i* and *j* using cosine similarity:


(8) $$D_{ij}^{w}=1-\frac{{\vec{f}}_{i}^{\;w}\cdot {\vec{f}}_{j}^{\;w}}{\left|{\vec{f}}_{i}^{\;w}||{\vec{f}}_{j}^{\;w}\right|},$$

where $\left|\vec{X}\right|$ indicates the magnitude of $\vec{X}$. A separate distance matrix is calculated for $\vec{e}{\;}_{s}^{\prime \;w}$:


(9) $$D_{ij}^{\prime w}=1-\frac{\vec{e}{\;}_{i}^{\prime \;w}\cdot \vec{e}{\;}_{j}^{\prime \;w}}{\left|\vec{e}{\;}_{i}^{\prime \;w}||\vec{e}{\;}_{j}^{\prime \;w}\right|},$$

and combined to form a final exponentiated distance matrix using element-wise exponentiation:


(10) $$K_{ij}^{w}=e^{\left(D_{ij}^{\prime w}+D_{ij}^{w}\right)}.$$

Finally, SCUDDO uses nonmetric multidimensional scaling (MDS) (Kruskal 1964) to transform the a×a matrix $K^{w}$ into a lower-dimensional representation, i.e. an a×p matrix where p<a, which preserves the distances in $K^{w}$. The MDS is followed by principal component analysis to further reduce the dimension to an a×q matrix $U^{w}$, where q<p (p=30 and q=5). This procedure is performed for the diagonal (w=0) and a given number of superdiagonals (w=ζ>0), and each set of dimensionality-reduced representations are concatenated, forming the a×(q(ζ+1)) matrix $R=U^{0},U^{1},\ldots ,U^{\zeta }$. R is then normalized feature-wise using the softmax function, $R_{ij}^{\prime }=e^{R_{ij}}/\sum_{\theta =1}^{a}e^{R_{\theta j}}$, and a distance matrix is constructed using the $L^{1}$ metric:


(11) $$S_{ij}=\sum_{\lambda =1}^{q(\zeta +1)}|R_{i\lambda }^{\prime }-R_{j\lambda }^{\prime }|.$$

Another round of dimensionality reduction is performed using MDS to reduce the dimension of S to the embedding size ϵ, which gives the a×ϵ matrix, V. Because there is no guarantee of convexity associated with each cluster when clustering single-cell Hi-C matrices, we use spectral decomposition before performing the clustering. In particular, SCUDDO transforms V into the similarity matrix, $A_{ij}=e^{-Z_{ij}^{2}}$, where $Z_{ij}=|{\vec{V}}_{i*}-{\vec{V}}_{j*}|$ and $V_{i*}$ is the vector consisting of all elements in the ith row of V. Next, we calculate the final a×(l−1) spectral embedding C, where the columns of C are the smallest l−1 eigenvectors (ignoring the trivial eigenvector) of the random-walk Laplacian matrix constructed from A using the Shi-Malik algorithm (Shi and Malik 2000) with log(a) nearest neighbors. We then input C and the number of labels *l* into a clustering algorithm, such as K-means++ (Arthur and Vassilvitskii 2007), which returns the predicted labels for each single-cell Hi-C map.

SCUDDO includes two tunable hyperparameters: ζ, the set of (super)diagonals, w=0,1,…,ζ used to construct the embedding vectors, and the dimension ϵ, to which V is reduced. By default, SCUDDO outputs two embeddings: C and V. Both ζ and ϵ are varied in Section 3 to study their effects on SCUDDO’s performance for each dataset. For all results in this study unless otherwise noted, we use C as the input into K-means++ and set ζ=25 and ϵ=5. Our results are not sensitive to the values of the dimensions *p* and *q*.

### 2.2 Benchmarking of single-cell Hi-C clustering algorithms

We focus our studies on four difficult-to-cluster datasets of single-cell Hi-C maps from recent benchmarking studies (Zhen et al. 2022, Ma et al. 2024, Plummer et al. 2025). In particular, we consider the Li et al. (2019) dataset (GEO ID: GSE119171) consisting of a=150 mouse embryonic stem cells that are separated into l=3 labels: “2i,” “Serum1,” and “Serum2,” the Flyamer et al. (2017) dataset (GEO ID: GSE80006) consisting of a=169 cells (154 after filtering) from developing mouse zygotes and oocytes with l=3 cell types: “Oocyte,” “ZygP,” and “ZygM” as labels, the Collombet et al. (2020) dataset (GEO ID: GSE129029) consisting of a=468 mouse embryo cells (466 after filtering) with labels that represent l=5 different cell stages: 1-cell, 2-cell, 4-cell, 8-cell, and 64-cell stages (we also study the full Collombet dataset of a=648 cells), and the Liu et al. (2023) dataset (hereafter denoted as “HiRES”; GEO ID: GSE223917) consisting of a=399 mouse brain cells with l=7 labels: “Astro,” “Ex1,” “Ex2,” “Ex3,” “In1,” “In2,” and “Oligo.” In previous benchmarking studies (Zhen et al. 2022), none of the eight tested methods for single-cell Hi-C map clustering achieved ARI or NMI ≥0.6 on the Collombet et al. dataset when using all 648 cells, and in another study (Zheng et al. 2022), none of the eight methods tested achieved an ARI >0.45 on the Li et al. dataset across any clustering algorithm (not just k-means). Similarly, when using a more lenient cell filter for the Flyamer et al. dataset, another benchmarking study (Plummer et al. 2025) found that none of the thirteen tested methods surpassed an average ARI (across several clustering algorithms not just k-means) of 0.6 and for the HiRES dataset none surpassed 0.32. For each dataset in the manuscript, we use 1 Mb bin sizes for the single-cell Hi-C maps, and re-bin those with higher resolution, as discussed in Zhou et al. (2019). For the Flyamer, Collombet, and HiRES datasets, we use the preprocessed single-cell Hi-C files from a previous benchmarking study (Plummer et al. 2025) (GEO ID: GSE305523) when applicable and for the Li dataset we use the dataset provided in another study (Zheng et al. 2022). We use the filtering procedure outlined in Plummer et al. (2025), which is based on the procedure outlined in Zhou et al. (2019). In particular, if the sum of all non-diagonal nonzero pairs of elements in the intrachromosomal Hi-C maps for a given cell is less than 5000, the data for this cell was not included in the analysis. Also, for each individual chromosome of size *x* for a cell, if the intrachromosomal Hi-C map for that chromosome has a sum of non-diagonal contacts that is less than *x*, all intrachromosomal Hi-C maps are not considered for that cell.

To select appropriate methods to benchmark for SCUDDO, we surveyed previous single-cell Hi-C clustering studies to identify the most consistently high-performing algorithms (Zheng et al. 2022, Ma et al. 2024, Plummer et al. 2025). In a recent comprehensive evaluation of 13 algorithms (Plummer et al. 2025), Higashi (Zhang et al. 2022), ScHiCluster (Zhou et al. 2019), and SnapATAC2 (Zhang et al. 2024) achieved the highest average ARI scores across eleven datasets at 1 Mb resolution. A separate study evaluating eight methods at 500 kb resolution (Ma et al. 2024) identified scVI-3D (Zheng et al. 2022) and scHiCluster as the top-performing methods on the Nagano et al. dataset, while Higashi, scVI-3D, and scHiCTools (Li et al. 2021) were among the top-performing methods on the Tan et al. dataset. Furthermore, a third study (Zheng et al. 2022) placed scVI-3D, Higashi, and scHiCluster within the top four algorithms (out of eight total) based on the median rank across four datasets. Drawing on these findings, we selected Higashi, scHiCluster, snapATAC2, scVI-3D, and InnerProduct (the highest-performing algorithm within the scHiCTools suite) as the methods for comparison with SCUDDO. These selections provide a well-rounded comparison that encompasses both statistically driven approaches (InnerProduct, scHiCluster) and training-intensive deep learning models (Higashi, scVI-3D). Importantly, all algorithms that we tested are unsupervised or self-supervised, and do not require labels for training. Other algorithms that require labels or significant pretraining are unable to cluster unlabeled single-cell Hi-C datasets, and thus they are not included in this manuscript. We ran each algorithm using its default hyperparameters; if none were specified, we utilized the parameters from a previous benchmarking study (Plummer et al. 2025). Because several methods are non-deterministic, we generated 10 independent embeddings for each algorithm. These embeddings were fed directly into K-means++ clustering without further processing to calculate mean ARI and NMI scores. Following prior benchmarking studies (Zhang et al. 2024, Plummer et al. 2025), latent embeddings for each method were capped at a maximum of 30 dimensions.

### 2.3 Metrics for clustering accuracy

To assess the accuracy of the predicted labels, we calculate the ARI (Hubert and Arabie 1985) and NMI (Vinh et al. 2010). Let $\Omega_{T}(s)$ and $\Omega_{G}(s)$ be functions that map each cell index *s* (from 1 to *a*) to the integers $l^{\prime }$ and $l^{\prime \prime }$ respectively, where $l^{\prime }$ is the ground truth label for cell *s* and $l^{\prime \prime }$ is the predicted label for cell *s*. We then define $P_{T}=\{X_{1},X_{2},\ldots X_{l}\}$ as the “ground-truth” label set, where $X_{l^{\prime }}$ denotes the set of cells such that $\Omega_{T}(s)=l^{\prime }$, and $P_{G}=\{Y_{1},Y_{2},\ldots Y_{l}\}$ as the “predicted” label set, where $Y_{l^{\prime \prime }}$ is the set of cells such that $\Omega_{G}(s)=l^{\prime \prime }$.

The ARI determines the similarity between the sets of cells with given ground truth and predicted labels:


(12) $$\text{ARI}=\frac{\sum_{i=1}^{l}\sum_{j=1}^{l}\left(\begin{array}{c} \beta_{ij}\\ 2 \end{array}\right)-(\sum_{i=1}^{l}\left(\begin{array}{c} \Gamma_{i}\\ 2 \end{array}\right)\sum_{j=1}^{l}\left(\begin{array}{c} \Delta_{j}\\ 2 \end{array}\right))/\left(\begin{array}{c} a\\ 2 \end{array}\right)}{\frac{1}{2}(\sum_{i=1}^{l}\left(\begin{array}{c} \Gamma_{i}\\ 2 \end{array}\right)+\sum_{j=1}^{l}\left(\begin{array}{c} \Delta_{j}\\ 2 \end{array}\right))-(\sum_{i=1}^{l}\left(\begin{array}{c} \Gamma_{i}\\ 2 \end{array}\right)\sum_{j=1}^{l}\left(\begin{array}{c} \Delta_{j}\\ 2 \end{array}\right))/\left(\begin{array}{c} a\\ 2 \end{array}\right)},$$

where $\beta_{ij}=[X_{i}\cap Y_{j}]$, ∩ is the intersection between two sets, *[X]* is the number of elements in set *X*, $\Gamma_{k}=\sum_{i=1}^{l}\beta_{ki}$, $\Delta_{k}=\sum_{j=1}^{l}\beta_{jk}$, and $\left(\begin{array}{c} m\\ n \end{array}\right)=\frac{m!}{n!(m-n)!}$. ARI=1 indicates a perfect match between $P_{T}$ and $P_{G}$, whereas ARI=0 indicates the match between $P_{T}$ and $P_{G}$ is no better than that achieved by random assignments in $P_{G}$.

We also quantify the accuracy of the clustering of the single-cell Hi-C maps using the NMI. NMI measures how much information can be learned about a given clustering by observing a different, but related clustering. NMI is defined as:


(13) $$\text{NMI}=\frac{\sum_{i=1}^{l}\sum_{j=1}^{l}H(i,j){\;\text{log}\;}_{2}\frac{H(i,j)}{H(i)H(j)}}{\sqrt{\left(-\sum_{i=1}^{l}H(i){\;\text{log}\;}_{2}H(i))(-\sum_{j=1}^{l}H(j){\;\text{log}\;}_{2}H(j)\right)}},$$

where $H(i)=\frac{\left[X_{i}\right]}{a}$, $H(j)=\frac{\left[Y_{j}\right]}{a}$, and $H(i,j)=\frac{\left[X_{i}\cap Y_{j}\right]}{a}$. 0≤NMI≤1, where NMI=1 indicates that $P_{T}=P_{G}$ and NMI=0 indicates that there is no correlation between $P_{T}$ and $P_{G}$. We calculate both ARI and NMI since they can differ for different-sized clusters: ARI is preferable when the sets in $P_{T}$ are similar in size, whereas NMI is preferable when the sets in $P_{T}$ are unbalanced. For all datasets and algorithms, we calculate the ARI and NMI after using the native embedding and K-means++ clustering.

## 3 Results

**Figure 3:**
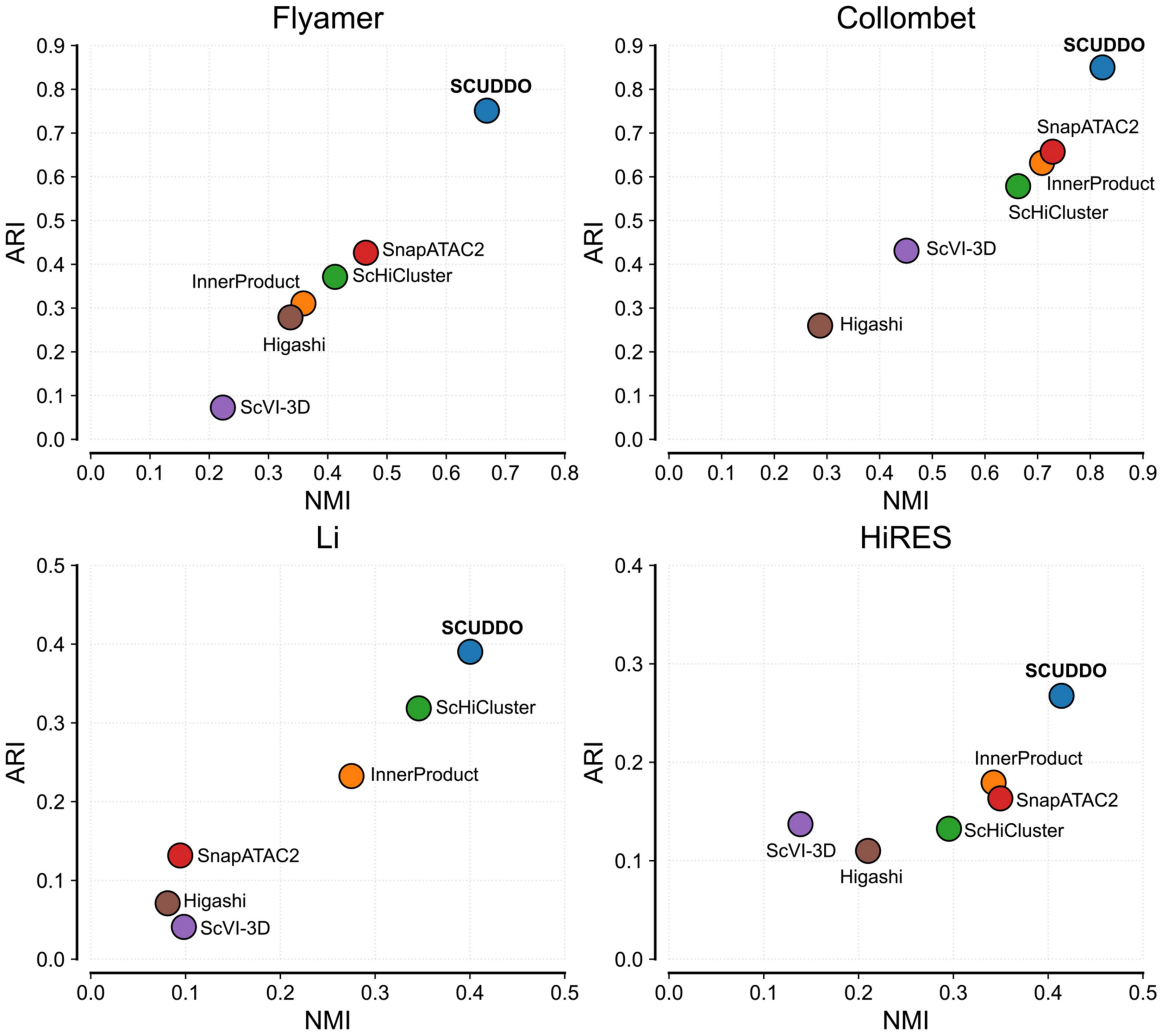
Accuracy of the six single-cell Hi-C map clustering algorithms [Higashi (Zhang et al. 2022) (brown), InnerProduct (Li et al. 2021) (orange), scHiCluster (Zhou et al. 2019) (green), snapATAC2 (Zhang et al. 2024) (red), scVI-3D (Zheng et al. 2022) (purple), and SCUDDO (blue)] on four difficult-to-cluster single-cell Hi-C datasets. We plot the ARI versus the NMI for each algorithm on the (top left) Flyamer et al. (2017), (bottom left) Li et al. (2019), (top right) Collombet et al. (2020), and (bottom right) HiRES (Liu et al. 2023) datasets.

We carried out single-cell Hi-C map clustering on four difficult-to-cluster datasets from Collombet et al. (2020), Flyamer et al. (2017), and Li et al. (2019), and on the HiRES dataset (Liu et al. 2023), using five current algorithms [Higashi (Zhang et al. 2022), InnerProduct (Li et al. 2021), scHiCluster (Zhou et al. 2019), snapATAC2 (Zhang et al. 2024), and scVI-3D (Zheng et al. 2022)] and compared the results to those obtained from SCUDDO. We plot ARI versus NMI for each dataset and algorithm in Fig. 3. Overall, SCUDDO outperforms the other five methods for all datasets tested. For the Li et al. dataset, we find a significant separation in accuracy between SCUDDO and the next most accurate method (scHiCluster). SCUDDO achieves an ARI∼NMI≈0.4, while scHiCluster achieves ARI≈0.31 and NMI≈0.33. For the Flyamer et al. dataset, we find that SCUDDO achieves an ARI≈0.75 and an NMI≈0.67, whereas the next most accurate method, snapATAC2, achieves an ARI≈0.43 and NMI≈0.46. Because the implementation of filtering we follow in this manuscript [from Plummer et al. (2025)] is more lenient (filtering on autosomes), we also ran benchmarks with a more restrictive Flyamer et al. dataset using only a=134 cells [from Zhen et al. (2022)] and found that SCUDDO still performed the best among the tested methods, achieving an ARI≈0.74 and NMI≈0.70 (scHiCluster achieves a notable ARI≈0.65 and NMI≈0.63). Interestingly, using a larger kernel size and standard deviation for the Gaussian kernel for SCUDDO allows SCUDDO to achieve ARI/NMI scores greater than 0.9 (see Fig. 1, available as supplementary data at *Bioinformatics* online). For the Collombet et al. dataset, we find that SCUDDO has ARI≈0.85 and NMI≈0.83, whereas the next most accurate methods, snapATAC2 and scHiCluster, achieve ARI∼NMI<0.65. Similar to the Flyamer et al. dataset, we also tested SCUDDO on a separate version of the Collombet et al. dataset [from Zhen et al. (2022)], consisting of more cells (a=648) and found again that SCUDDO performs the best among the tested methods scoring an ARI∼NMI≈0.62 whereas no other method surpassed ARI/NMI scores of 0.5. Lastly, for the HiRES dataset, we again find that SCUDDO outperforms all tested methods, scoring an ARI≈0.28 and NMI≈0.41 whereas the next best methods, InnerProduct and snapATAC2, score ARI≈0.18 and NMI≈0.34. Interestingly, when setting the ζ hyperparameter to lower values (i.e. sampling fewer superdiagonals), SCUDDO achieves ARI scores greater than 0.45, suggesting that higher superdiagonals potentially degrade clustering ability. In addition to preserving major cell-type lineages in the HiRES dataset, we additionally tested whether SCUDDO could simultaneously capture structural features during the cell cycle for the HiRES dataset. To ensure that this separation was an inherent property of SCUDDO’s high-dimensional space and not an artifact of a 2D non-linear projection, we performed a k-nearest neighbors (k-NN, *k* = 5) analysis directly on the raw, unmanipulated SCUDDO embeddings. We found that a cell’s closest high-dimensional structural neighbors were highly predictive of its continuous mitotic percentage and short-range contact fraction. The mean mitotic score of a cell’s local neighborhood strongly and significantly correlated with the cell’s actual score (Pearson *R* = 0.8) and the same held true for short-range contact fraction (Pearson *R* = 0.85). This effect was weaker (Pearson *R* = 0.34) when looking at nearby neighbors for similar replication scores, suggesting that SCUDDO is better able to capture some cell cycle features than others.

**Figure 4:**
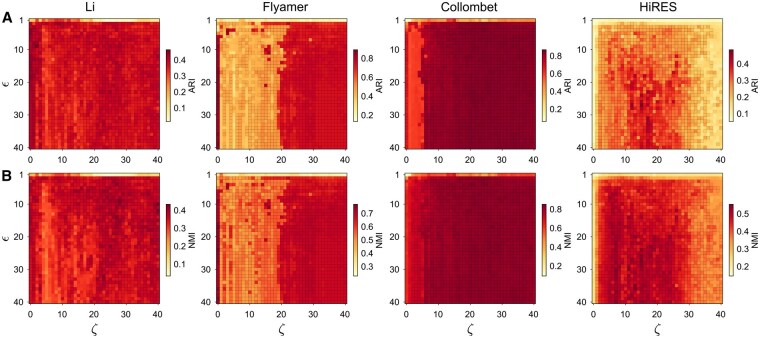
The clustering accuracy [ARI in (A) and NMI in (B)] for the SCUDDO algorithm for each of the four datasets, Li et al. (2019), Flyamer et al. (2017), Collombet et al. (2020), and the HiRES dataset (Liu et al. 2023), plotted as a function of the number of sampled superdiagonals ζ and the embedding dimension ϵ prior to spectral decomposition. The pixels in the heatmap are outlined when the ARI or NMI for SCUDDO exceed those of the next best performing method. The default values for SCUDDO are ζ=25 and ϵ=5.

We next show that SCUDDO can accurately embed single-cell Hi-C maps using a reduced amount of information for already highly sparse single-cell Hi-C maps, surpassing the accuracy of previous algorithms using fewer intrachromosomal matrices for each cell, as well as fewer sampled superdiagonals for each matrix. We study the performance of SCUDDO after restricting the single-cell Hi-C data available to it in two ways: first by varying the hyperparameter ζ for the number of superdiagonals to sample for each intrachromosomal Hi-C map, as well as varying the hyperparameter ϵ for the embedding dimension of V. In Fig. 4, we show heatmaps of the ARI and NMI for the SCUDDO algorithm, while varying 0≤ζ≤40 and 1≤ϵ≤40. The outlined pixels in the ζ-ϵ plane indicate ARI or NMI values for the SCUDDO algorithm that exceed those for all other methods (when they use all of the available single-cell Hi-C data).

For the Li et al. dataset, we show in the leftmost column of Fig. 4A and B that for ≈93% and 72% of the ζ-ϵ plane, SCUDDO outperforms all methods in ARI and NMI, respectively. In particular, across all ζ values, SCUDDO’s mean ARI (over all ϵ) exceeds the next best method. Similarly, when ϵ>1, SCUDDO gives mean ARI values over all ζ that exceed the values for all other methods. Even in regimes with low superdiagonal sampling and embedding dimension, SCUDDO can obtain ARI values that surpass the next best method (e.g. ζ=0,ϵ=2). The maximum ARI and NMI for clustering the Li et al. dataset using SCUDDO in the sampled hyperparameter space were ≈0.47 and ≈0.44, respectively.

On the Flyamer et al. dataset, SCUDDO outperforms the other methods again over a large region of the hyperparameters ζ and ϵ, as shown in Fig. 4A and B, with ≈80% and≈95% of (ζ,ϵ) input pairs into SCUDDO resulting in ARI and NMI scores that surpassed the next best method’s ARI and NMI scores, respectively. We find that SCUDDO requires generally higher ζ values to perform ideally on the Flyamer et al. dataset, showing a sudden improvement around ζ=20 for both ARI and NMI. The exception is when ζ=0, where a strong band exists once ϵ≥6. Across all ζ and ϵ pairs, SCUDDO achieves a maximum ARI of ≈0.89 and NMI of ≈0.77, both surprisingly when ϵ=17 and ζ=0.

For the Collombet et al. dataset, SCUDDO outperforms the next best method in ARI and NMI over ≈87% and ≈90% of the ζ-ϵ plane. For ζ>5, the mean ARI and NMI across all ϵ values for SCUDDO is larger than the best ARI and NMI from all other tested algorithms. Similarly, SCUDDO outperforms all the other methods in mean ARI and NMI when ϵ>1 (across all ζ). Across the sampled hyperparameters, we find that the maximum ARI and NMI are ≈0.87 and ≈0.85, respectively.

On the HiRES dataset, SCUDDO performs ideally when ζ<30 and ϵ>20. Despite this, SCUDDO performs very well over a large region of the hyperparameters ζ and ϵ, with ≈79% and≈87% of (ζ,ϵ) input pairs into SCUDDO resulting in ARI and NMI scores that surpassed the next best method’s ARI and NMI scores, respectively. For ζ>2, the mean ARI and NMI across all ϵ values for SCUDDO is larger than the best ARI and NMI from all other tested algorithms. Similarly, SCUDDO outperforms all the other methods in mean ARI and NMI when ϵ>2 (across all ζ) for the HiRES dataset. Across all ζ and ϵ pairs, SCUDDO achieves a maximum ARI of ≈0.49 and NMI of ≈0.55, which is a significant improvement in ARI and NMI from the default hyperparameter values.

Previous single-cell clustering algorithms often require a large number of dimensions (ϵ≳100) to achieve reasonable clustering accuracy on single-cell Hi-C maps (Zhou et al. 2019). In addition, the ARI and NMI can possess large fluctuations as a function of the embedding dimension and depend strongly on the specific dimensionality reduction technique that is implemented, for instance with some methods requiring specific dimensionality reduction techniques to be competitive (Zhang et al. 2022). In contrast, we have shown that the ARI and NMI scores for SCUDDO are large at both very low embedding dimensions and when sampling only a few superdiagonals. This result is true even when we treat ϵ as the final embedding dimension of the output for SCUDDO, despite the fact that SCUDDO always outputs a l−1 dimensional embedding, where l<ϵ for all datasets except for HiRES. In addition, we find that SCUDDO does not depend sensitively on the specific dimensionality reduction technique. For instance on the Collombet et al. dataset, SCUDDO performs roughly equivalently when V is embedded spectrally (i.e. the default embedding) (ARI≈0.85), embedded using UMAP (McInnes et al. 2018) (ARI≈0.84), embedded using t-SNE (Hinton and Roweis 2002) (ARI≈0.85), and when there is no further dimensionality reduction and using V directly (ARI≈0.78). For detailed UMAP and t-SNE embeddings of SCUDDO for all datasets, see Fig. 4, available as supplementary data at *Bioinformatics* online. While the default values for ζ and ϵ for SCUDDO were not optimized to the four selected datasets, the default parameters give excellent results for ARI and NMI for these datasets. However, there are (ζ,ϵ) pairs that give superior performance across all datasets in this manuscript.

**Figure 5:**
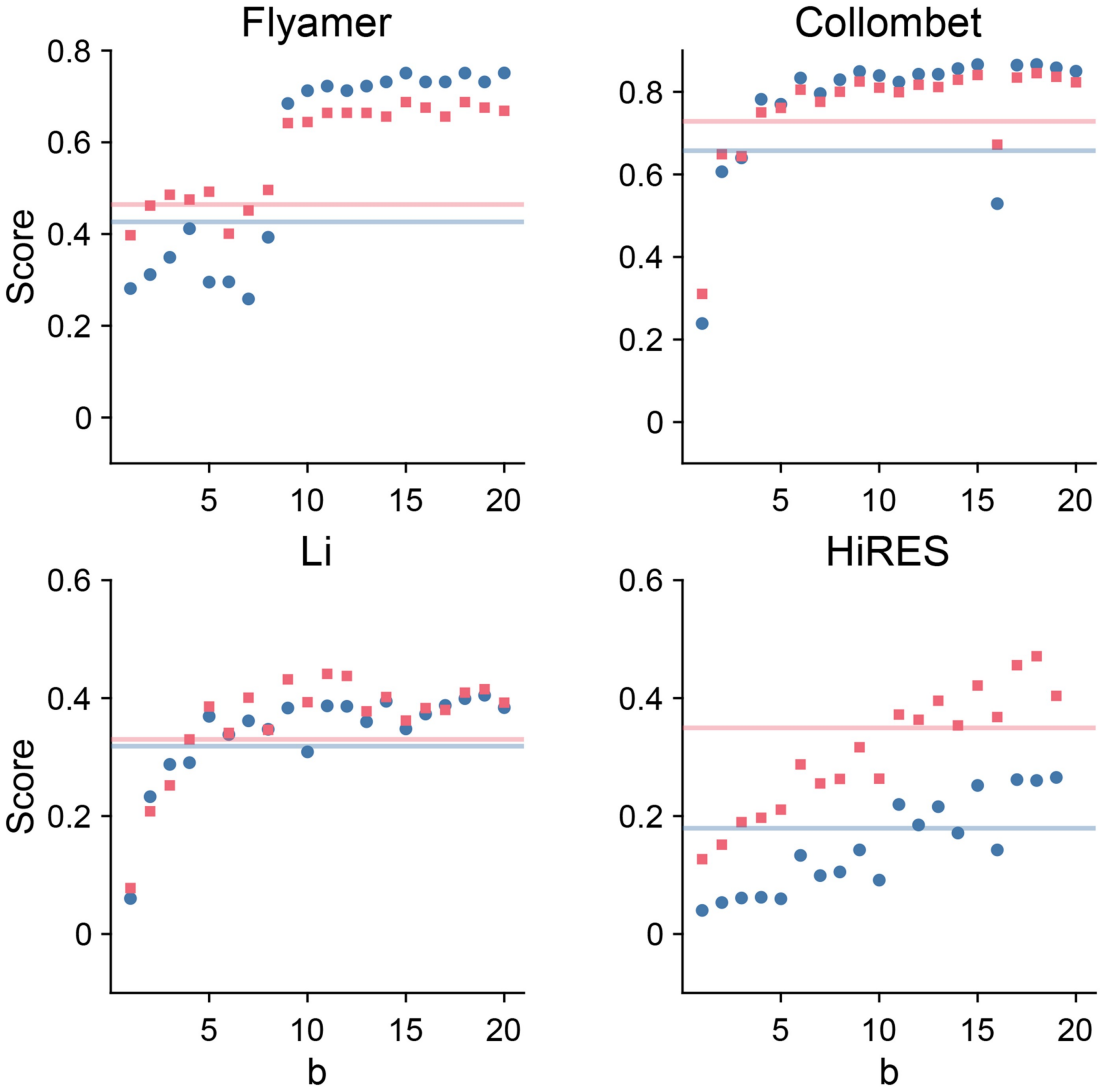
The clustering accuracy, ARI (red squares) and NMI (blue circles), for the SCUDDO algorithm plotted as a function of the number of sampled chromosomes *b* for the four datasets: (top left) Flyamer et al. (2017), (bottom left) Li et al. (2019), (top right) Collombet et al. (2020), and (bottom right) the HiRES dataset (Liu et al. 2023). The faint horizontal red and blue lines represent the maximum values of ARI and NMI achieved by the other methods. The next best ARI/NMI scores do not have to come from one method.

In Fig. 5, we calculate ARI and NMI for the four datasets versus the number of chromosomes used by SCUDDO. For these calculations, we sample all chromosomes with an index less than or equal to *b*, for instance, if we set b=4, SCUDDO samples only chromosomes 1, 2, 3, and 4. For the Li et al. and Collombet et al. datasets, we find that the ARI and NMI for the SCUDDO algorithm first exceed those for the next best method when b≳5, respectively (with the exception of Collombet showing a dip at b=16). For the Flyamer et al. dataset, we find that when b≳10 there is a jump in SCUDDO ARI/NMI scores, past which the ARI/NMI is mostly stable and higher than the next best ARI/NMI. The HiRES dataset requires the most chromosomes to sample, with SCUDDO only achieving state-of-the-art performance when b≳11 and even then with some dips in performance at b=14,16.

## 4 Discussion

In this article, we develop a novel algorithm, SCUDDO, to determine a low-dimensional representation and then cluster single-cell Hi-C maps. We focused on four difficult-to-cluster single-cell Hi-C map datasets, where the datasets include ground-truth labels for each single-cell Hi-C map. We compared the ARI and NMI metrics for clustering accuracy from SCUDDO to those from five other clustering algorithms that were the most accurate in previous single-cell Hi-C map clustering benchmarking studies (Zhen et al. 2022, Ma et al. 2024, Plummer et al. 2025). SCUDDO is more accurate in all cases in terms of both ARI and NMI compared to the other methods for all datasets. We also find that SCUDDO can accurately cluster single-cell Hi-C maps using a fraction of the intrachromosomal Hi-C maps and their superdiagonals, as well as fewer embedding dimensions.

SCUDDO has several advantages over other existing methods for clustering single-cell Hi-C maps. First, SCUDDO uses, to our knowledge, a new imputation technique for single-cell Hi-C maps, calculating a graph kernel, then smoothing over local neighborhood features in individual intrachromosomal matrices using a Gaussian kernel, and finally using matrix exponentiation. We find that this technique readily improves the accuracy for clustering, since it allows both short-range and long-range information transfer within an intrachromosomal Hi-C map (for a detailed ablation analysis of SCUDDO, see Fig. 2, available as supplementary data at *Bioinformatics* online). SCUDDO also mainly uses PCA and MDS for dimensionality reduction, both of which are much more interpretable than dimensionality reduction techniques like UMAP and t-SNE or using complex inscrutable networks that require training to find features. SCUDDO is also fast and scalable (Fig. 3, available as supplementary data at *Bioinformatics* online) and is highly parallelizable since each (super)diagonal matrix can be processed independently in parallel. Additionally, we provide a version of SCUDDO (“fast SCUDDO”) that uses several computational shortcuts including a truncated Taylor series expansion for the matrix exponential, classical MDS instead of non-classical MDS, and a Neumann series approximation for the graph kernel to achieve extremely fast runtime (≈5000 cells were run in <5 minutes) at the expense of some accuracy (a mean loss in ARI of ≈0.05).

The SCUDDO algorithm combines two key features: the diffused (normalized) superdiagonals of each single-cell Hi-C map and the trinarized differences along the diffused superdiagonals. The algorithm then calculates the angles between these features for each superdiagonal (using cosine similarity) and performs several steps of dimensionality reduction to achieve a final embedding. We find that for some datasets both features are necessary to achieve the best clustering accuracy, e.g. for the HiRES dataset using only one feature scores a maximum ARI of only ≈0.15. While the details of the features and SCUDDO algorithm are easy to interpret mathematically, the biophysical interpretation of these features is less clear. For example, it is unclear whether the diffusion and smoothing steps used by SCUDDO have a clear biophysical interpretation.

While our results show that SCUDDO maintains state-of-the-art clustering performance when using both high-dimensional latent vectors and 2D projections (e.g. UMAP and t-SNE; see Section 3 and Fig. 4, available as supplementary data at *Bioinformatics* online), the preservation of global higher-order structures in 2D may not hold across all single-cell Hi-C datasets due to topological distortions with further dimensionality reduction. Thus, utilizing these lower-dimensional visualizations for critical applications such as aligning modalities, defining cell-type boundaries, or aggregating cells for pseudo-bulk analysis can introduce false associations if the projection distorts the underlying manifold. We therefore recommend that users exercise caution in making inferences from lower-dimensional features like neighbors or distances and when possible, to use the higher-dimensional latent embeddings rather than relying solely on visual proximity in 2D space.

Interesting future studies involve coupling molecular dynamics simulations of polymer models of chromosomes (Sun et al. 2021) with the SCUDDO algorithm to further improve clustering accuracy and to better understand the biophysical mechanisms that support the efficacy of the methods used in the SCUDDO algorithm. In addition, the SCUDDO algorithm can be applied to synthetic datasets with ground-truth labels and tunable noise and sparsity, as well as to experimental datasets without labels to predict cell fate.

## Supplementary Material

btag284_Supplementary_Data
