# Supplementary material for: SCUDDO: an unsupervised clustering algorithm for single-cell Hi-C maps using diagonal diffusion operators
Source: Bioinformatics. 2026 May 11;42(5):btag284. doi: 10.1093/bioinformatics/btag284 (PMC13202327; doi:10.1093/bioinformatics/btag284)
Supplement: btag284_Supplementary_Data [file btag284_supplementary_data.pdf]

Supplementary Material:

SCUDDO: An unsupervised clustering algorithm for single-cell Hi-C maps using diagonal diffusion operators

Luka Maisuradze, Mark D. Shattuck, and Corey S. O'Hern

Supplementary Figures

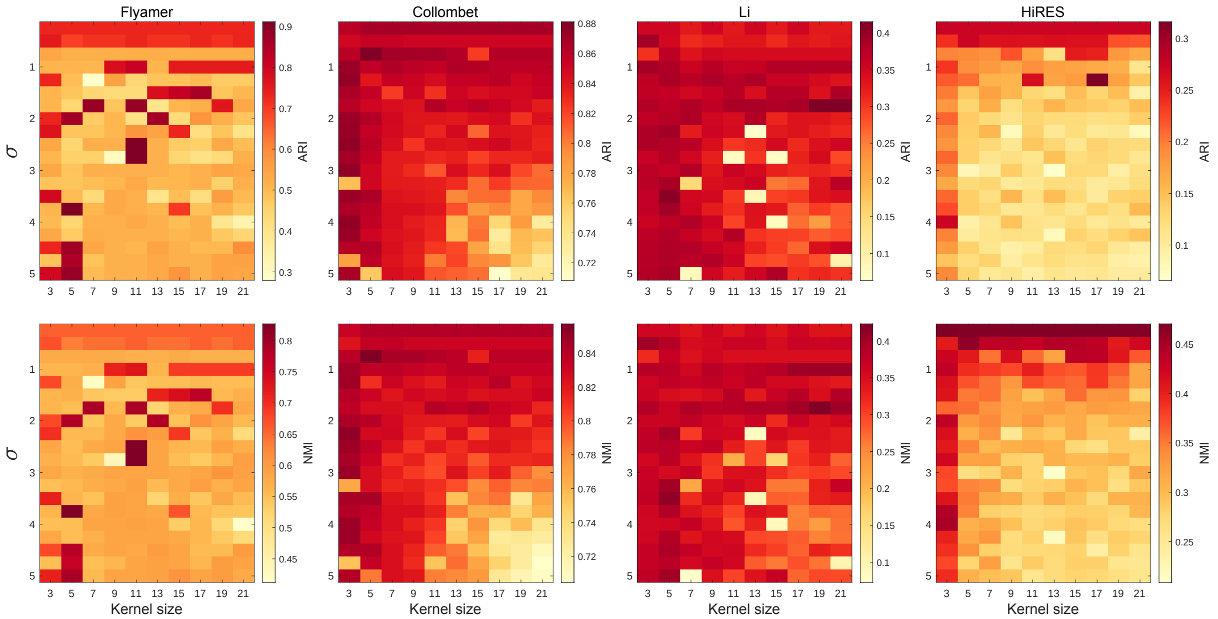

Figure S1: SCUDDO ARI (top row) and NMI (bottom row) scores for each dataset (columns) across a sweep of σ (from 0.25 to 5) and kernel size (from 3 to 21). While the Collombet and Li datasets are relatively uniform in ARI/NMI (with performance decrease with very high kernel size and σ), the HiRES and Flyamer datasets show faster decreases after $\sigma > 1$ and kernel size > 3.

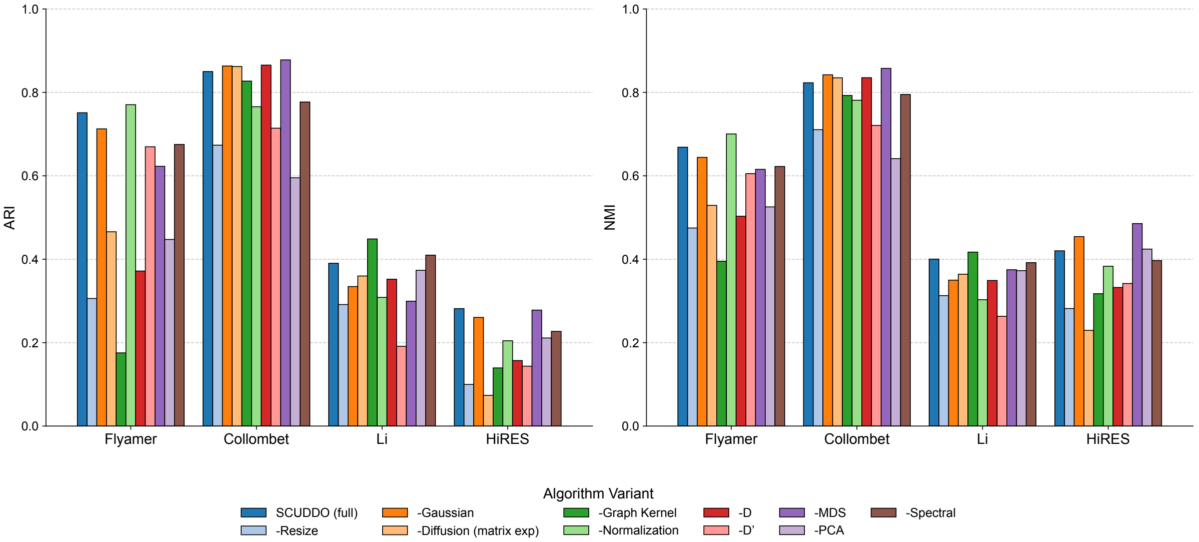

Figure S2: ARI (left plot) and NMI (right plot) scores for different ablations of SCUDDO across the four datasets considered in the manuscript. Each ablation is denoted with a '-', e.g. "-Gaussian" is SCUDDO with the removal of the Gaussian kernel step in the imputation process.

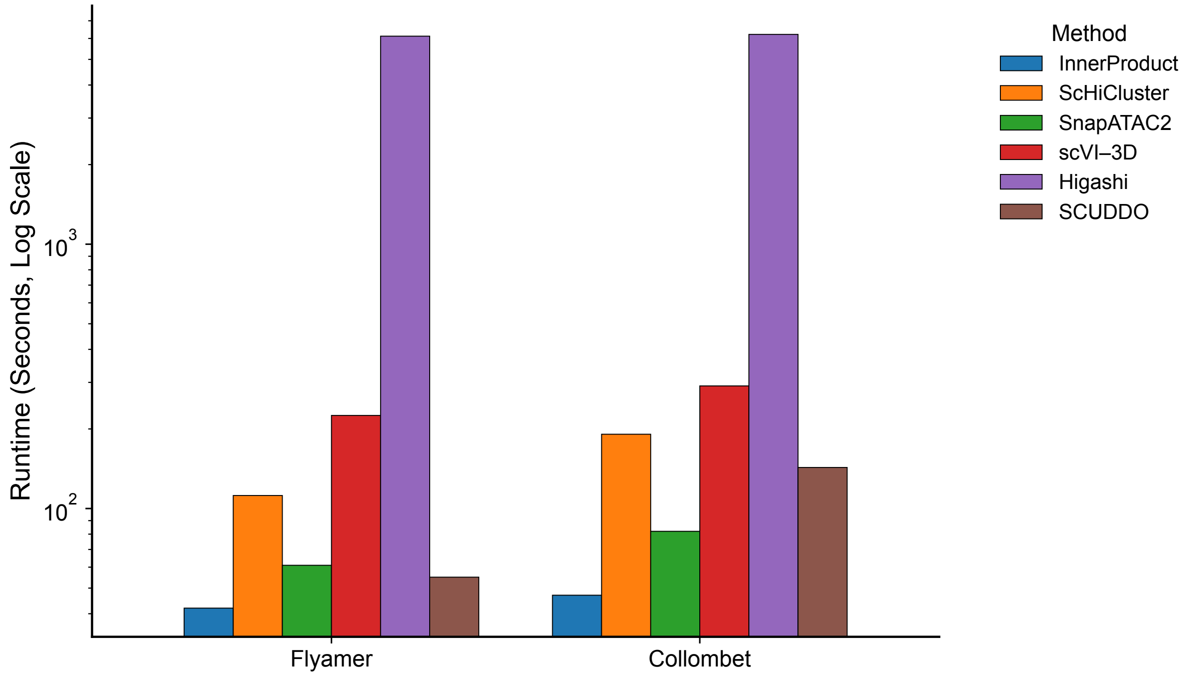

Figure S3: The runtime in seconds (log scale) of each algorithm studied in the manuscript for the Flyamer (left) and Collombet (right) datasets, both upsampled to 500kb from 1Mb to test larger single-cell Hi-C matrices.

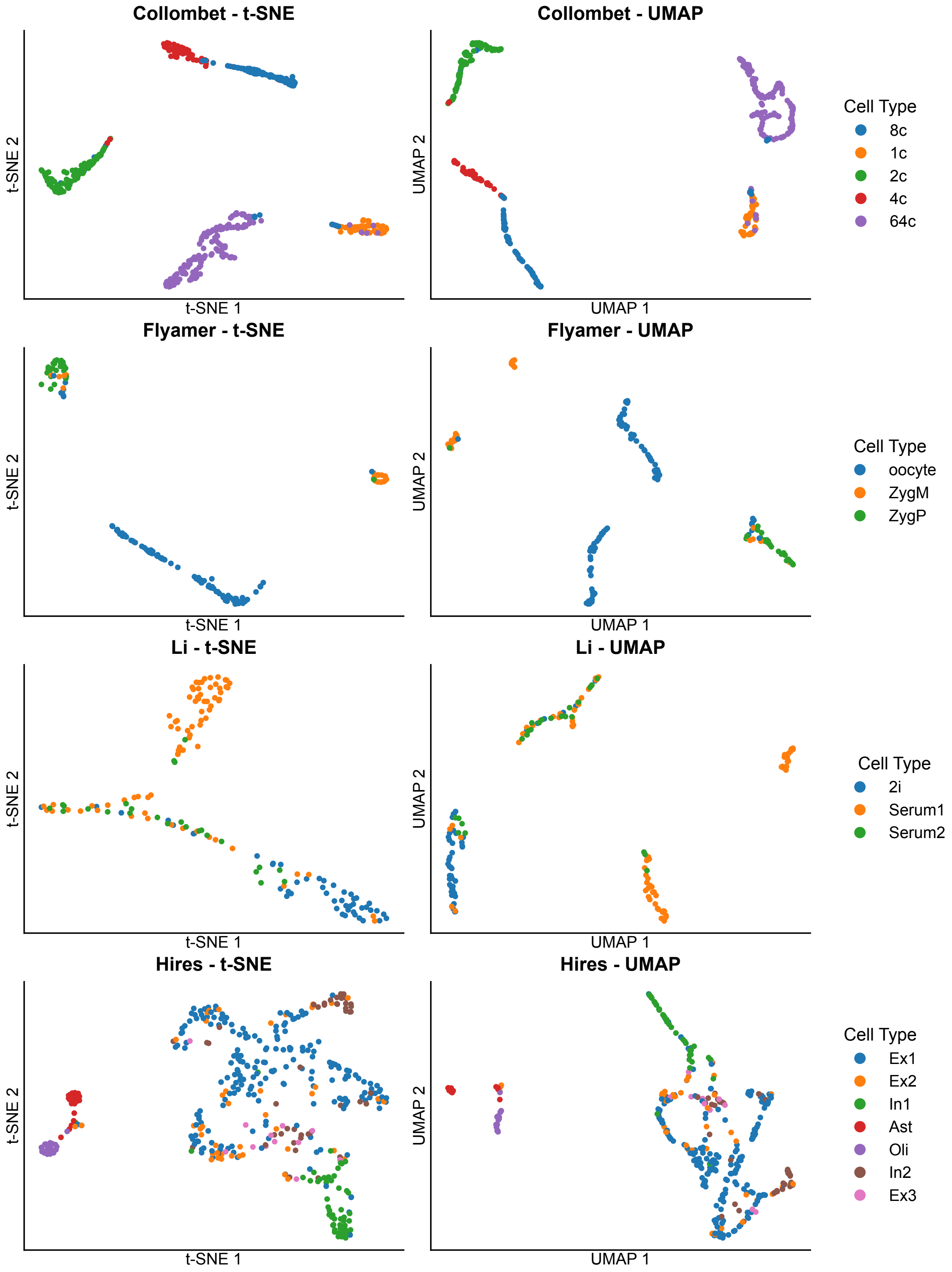

Figure S4: Two-dimensional projections of SCUDDO embeddings. t-SNE (left column) and UMAP (right column) visualizations of the latent spaces generated by SCUDDO for the single-cell Hi-C datasets. Individual points represent single cells and are colored according to their annotated cell-type labels. Both dimensionality reduction techniques were applied using their default hyperparameters (e.g., UMAP n neighbors=15).
